# CBCT Image Superimposition for Longitudinal Monitoring of Mandibular Cyst Healing: A Technical Note

**DOI:** 10.1002/cre2.70292

**Published:** 2026-02-18

**Authors:** Francesco Fanelli, Angela Tisci, Khrystyna Zhurakivska, Giuseppe Troiano

**Affiliations:** ^1^ Department of Clinical and Experimental Medicine University of Foggia Foggia Italy; ^2^ Department of Medicine and Surgery LUM University Casamassima Italy

**Keywords:** cone‐beam computed tomography, follow‐up studies, image registration techniques, jaw cysts

## Abstract

**Objectives:**

To evaluate the effectiveness of spatial superimposition of cone‐beam computed tomography (CBCT) scans acquired at baseline and 6‐month follow‐up for monitoring bone healing in mandibular cystic lesions, aiming to reduce variability in conventional volumetric comparisons and enhance accuracy, reproducibility, and spatial fidelity of radiographic assessments.

**Materials and Methods:**

Two CBCT scans of a single patient (baseline, T0; follow‐up, T1) were imported into 3D Slicer v5.8.0. The mandible was isolated by manually cropping a region of interest, and the cystic lesion was segmented semi‐automatically using the Grow from Seeds tool. A rigid six‐degree‐of‐freedom registration aligned T1 to T0; the resulting transformation matrix was applied to the T1 segmentation to enable direct voxel‐wise comparison. Lesion volumes were measured, and spatial subtraction analysis quantified the resorbed area.

**Results:**

Lesion volume decreased from 1841.64 mm^3^ at T0 to 1362.62 mm^3^ at T1, corresponding to an absolute reduction of 479.02 mm^3^ (26.0%). The subtraction mask accurately localized regressed voxels, confirming both the magnitude and spatial distribution of bone healing.

**Conclusions:**

Potential limitations related to CBCT artifacts and the need for operator‐dependent manual steps should be considered when interpreting the results. Overall, CBCT superimposition with semi‐automatic segmentation provides an objective, consistent, and anatomically precise approach for monitoring mandibular cyst regression and may represent a useful tool to support conservative management strategies.

## 1 Introduction

Bone lesions of the jaws encompass a wide range of pathological conditions, including odontogenic cysts, tumors, inflammatory processes, and traumatic or surgical defects, all of which can significantly affect the oral‐maxillofacial complex (Vered and Wright 2022). These lesions, depending on their nature, may cause symptoms such as pain, swelling, facial asymmetry, and impairment of essential functions, including mastication, speech, and esthetics (Du et al. 2024). Medically, accurate diagnosis, management, and meticulous follow‐up of these lesions are crucial not only for resolving immediate clinical symptoms but also for preventing long‐term complications such as pathological fractures, infection recurrence, or malignant transformation (Tian et al. 2024).

Therapeutic management typically involves surgical interventions aimed at removing the lesion and, when necessary, reconstructing the residual bony defect (Safadi et al. 2020). Surgical procedures may range from simple curettage or enucleation of cystic lesions to extensive resections and bone grafting in more severe or complex cases. More conservative techniques such as marsupialization and/or decompression are sometimes used as the first choice and/or preliminary approach to reduce patient morbidity (Castro‐Nunez 2016). Regardless of the surgical approach, monitoring the postoperative healing and remodeling of bone is essential for evaluating treatment success and planning further interventions if necessary (La Rosa et al. 2024).

Clinically, the assessment of healing involves monitoring patient symptoms, evaluating soft tissue changes, and assessing the integrity and function of the affected region. However, radiographic evaluation provides indispensable objective data regarding the quality, quantity, and progression of bone regeneration. Cone‐beam computed tomography (CBCT) has emerged as a leading imaging modality due to its ability to provide accurate three‐dimensional (3D) visualization of osseous structures with relatively low radiation exposure and high spatial resolution (Etöz et al. 2021).

Traditional methods for radiographic evaluation of jawbone lesions typically rely on independent volumetric segmentation of lesions at various postoperative time points, comparing lesion volumes to assess healing indirectly (Shi et al. 2022). While effective, these methods may introduce variability due to manual segmentation and lack explicit spatial alignment between follow‐up examinations.

In this context, the development of advanced techniques that allow direct superimposition of CBCT scans taken at different time points represents an important methodological refinement. Such an approach can significantly enhance the accuracy, reproducibility, and spatial fidelity of healing assessments, providing clinicians with more precise, objective, and clinically relevant information about jawbone regeneration processes.

## 2 Materials and Methods

The lesion was diagnosed clinically and radiographically as an inflammatory radicular cyst associated with the mandibular right canine and first premolar (teeth 43 and 44). Nonsurgical endodontic treatment was performed on both teeth, followed by conservative management and radiographic follow‐up at 6 months. Two CBCT scans of a single patient were acquired 6 months apart, baseline (T0) and follow‐up (T1), using the same machine settings: isotropic voxel size 0.25 mm, matrix 600 × 600 × 312 voxels. The DICOM volumes were imported into 3D Slicer v5.8.0 (Fedorov et al. 2012) for all subsequent processing. The original CBCT volumes used for registration are shown in Figures S1 (T0) and S2 (T1).

### 2.1 Mandible Isolation and Resampling

To restrict the analysis to a stable anatomical region and reduce computational burden, the mandible was isolated using the Crop Volume module. For each scan, a 3D region of interest (ROI) was manually defined in the axial, sagittal, and coronal planes to encompass the entire mandibular bone. The cropped volumes were subsequently resampled to the original voxel size (0.25 mm) using linear interpolation to ensure spatial consistency between T0 and T1, resulting in the data sets cbct_T0_mand_crop and cbct_T1_mand_crop.

### 2.2 Lesion Segmentation

Lesion segmentation was performed using the semi‐automatic *Grow from Seeds* algorithm implemented in 3D Slicer. To ensure reproducibility between time points, the same intensity threshold and segmentation parameters were applied to both baseline (T0) and follow‐up (T1) CBCT scans. Threshold selection was guided by visual inspection of lesion boundaries and kept constant across scans, in accordance with previously published quantitative CBCT approaches (Lewin et al. 2019).

**Figure 1:**
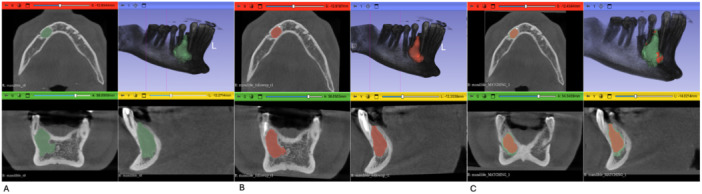
Segmentation and image registration of the cystic lesion. (A) Baseline scan (T0): segmentation of the cystic lesion (green) using the “Grow from Seeds” tool. (B) Follow‐up scan (T1): segmentation of the cystic lesion (red) using the same method. (C) Spatial registration: the T1 segmentation (red) is rigidly registered and overlaid on the T0 anatomical reference (green), enabling direct visual and spatial comparison.

For both time points, two seed regions were defined: one placed within the lesion (foreground) and one within the surrounding healthy bone or tissue (background). These seeds guided a region‐growing algorithm that expanded voxels with similar intensity values. The resulting contours were inspected in axial, sagittal, and coronal planes, and seed placement was iteratively refined until accurate delineation of the lesion boundaries was achieved (Figure 1A,B). Final binary masks were exported as *Cyst_T0* and *Cyst_T1*.

### 2.3 Rigid Registration

**Figure 2:**
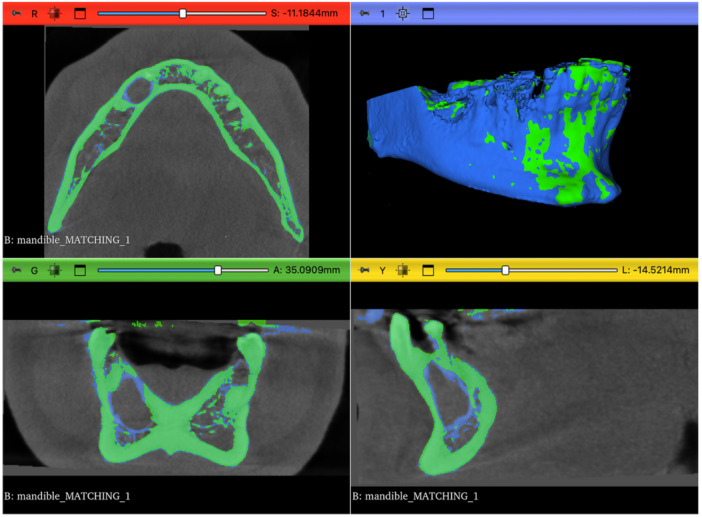
Visual assessment of CBCT co‐registration between baseline (T0, green) and follow‐up (T1, blue) mandibular volumes in axial, coronal, and sagittal planes.

To enable direct voxel‐wise comparison between baseline (T0) and follow‐up (T1), the cropped follow‐up volume (cbct_T1_mand_crop) was rigidly registered to the baseline volume (cbct_T0_mand_crop) using the General Registration (BRAINS) module. Registration was performed using a six‐degree‐of‐freedom (6‐DOF) rigid transformation (three translations and three rotations) driven by a mutual information similarity metric. Mutual information quantified the statistical dependence between voxel intensity distributions of the two CBCT scans, allowing robust alignment despite minor anatomical changes and potential intensity differences. The resulting transformation matrix (TRANSFORM_T1_TO) was applied to the follow‐up lesion segmentation and hardened using the Transforms module, ensuring that the T1 segmentation shared the same coordinate system as the baseline scan and enabling accurate voxel‐wise comparison. (Figure 1C). Visual assessment of registration quality was performed by inspecting the superimposition of baseline and follow‐up CBCT volumes in axial, sagittal, and coronal planes (Figure 2).

### 2.4 Quantitative and Spatial Analysis

Absolute lesion volumes were measured using the *Segment Statistics* module in 3D Slicer, which computes voxel count and multiplies it by voxel volume to obtain measurements in mm^3^. Quantitative results for *Cyst_T0* and the transformed *Cyst_T1* segmentations were exported in CSV format. To visualize spatial changes, the *Logical Operators* module in 3D Slicer was used to subtract the T1 mask from the T0 mask, generating a difference mask representing voxels present only at baseline. Volume, surface area, and voxel count of this difference mask were computed within 3D Slicer. The resulting outputs were subsequently processed using Python code executed within the 3D Slicer Python environment for data handling and visualization (pandas, matplotlib). This integrated workflow enabled both objective volumetric comparison and spatial visualization of lesion regression over the 6‐month follow‐up period.

## 3 Results

**Figure 3:**
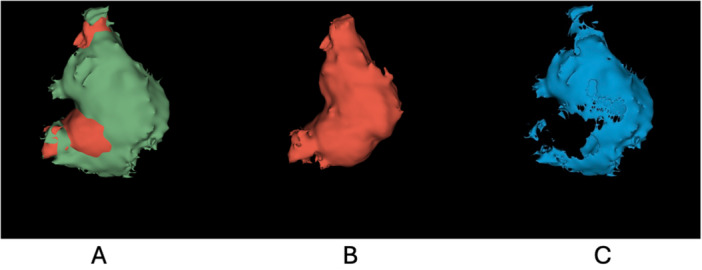
Three‐dimensional volumetric comparison and subtraction analysis of the cystic lesion: (A) (green + red): Superimposition of the baseline (T0, green) and follow‐up (T1, red) segmentations, showing lesion regression. (B) (red): Final aligned segmentation of the lesion at follow‐up (T1). (C) (blue): Subtraction map highlighting the resorbed portion of the lesion (voxels present at T0 only).

Volumetric analysis demonstrated a clear reduction in lesion size over the 6‐month follow‐up period. At baseline (T0), the cyst volume measured 1841.64 mm^3^, decreasing to 1362.62 mm^3^ at follow‐up (T1), corresponding to an absolute reduction of approximately 479.02 mm^3^ (26.0%; Table 1). The subtraction mask (T0–T1) consistently identified regressed voxels with a total volume corresponding to the observed volumetric reduction (Figure 3C). Additional quantitative descriptors of the difference mask, including surface area, voxel count, and intensity statistics, are reported in Table 1. Spatial evaluation across axial, sagittal, and coronal planes (Figure 3A) demonstrated consistent anatomical alignment of the T1 segmentation relative to the T0 anatomy, with no evident misalignment on visual inspection. The subtraction map (Figure 3C) localized regions of volumetric regression, while the aligned follow‐up segmentation is shown in Figure 3B. Together, these volumetric and spatial findings support objective assessment of mandibular cyst regression following conservative management.

**Table 1:** Morphometric and intensity‐based metrics compare the cyst at baseline (Cyst_T0) and 6‐month follow‐up (Cyst_T1).

Segment	Voxel count (LM)	Volume mm^3^ (LM)	Volume cm^3^ (LM)	Voxel count (SV)	Volume mm^3^ (SV)	Volume cm^3^ (SV)	Minimum	Maximum	Mean	Standard deviation	Percentile 5	Percentile 95	Median	Surface mm^2^	Volume mm^3^ (CS)	Volume cm^3^ (CS)
**Cyst_T0**	119,280	1863.75	1.86375	119,280	1863.75	1.86375	−584	992	100.819	143.992	−56	388	68	1042.02	1841.64	1.84164
**Cyst_T1**	87,675	1369.92	1.36992	86,832	1356.75	1.35675	−564	1220	113.833	151.494	−53	434	77	793.202	1362.62	1.36262

*Note:* Voxel counts and volumes are derived both from binary label‐map segmentations and from closed‐surface (marching cubes) reconstructions. Intensity statistics, minimum, maximum, mean, standard deviation, median, and 5th/95th percentiles, were calculated from the scalar volume. The observed decreases in voxel count, volume, and surface area between T0 and T1 confirm clear lesion regression.

## 4 Discussion

The CBCT image superimposition technique presented in this study provides significant advantages over traditional radiographic methods for assessing jawbone lesion healing (Shi et al. 2022; Tahmasbi‐Arashlow et al. 2022). Conventional methods often rely on independent manual segmentation of lesions at different time points, which may be associated with operator‐dependent variability (Tian et al. 2024; Ku et al. 2022). By enabling direct voxel‐wise comparison of CBCT scans acquired longitudinally, image superimposition can facilitate a more consistent spatial assessment of lesion changes over time. While inter‐ and intra‐observer variability were not formally quantified in the present study, the use of a common spatial reference frame may help reduce segmentation‐related inconsistencies and improve the objectivity of radiographic evaluation.

Moreover, precise spatial alignment between pre‐ and post‐treatment images allows for more accurate detection of subtle spatial changes and bone remodeling dynamics, details that might be missed using simple independent volumetric analyses. This feature is particularly relevant clinically, especially for conservative treatments such as marsupialization or decompression, where careful monitoring of gradual lesion resolution is critical for decision‐making regarding subsequent interventions or adjustments to the therapeutic plan (Berretta et al. 2021). The capability to objectively quantify volumetric reduction, as demonstrated by the observed 26% decrease in lesion volume over a 6‐month period, further underscores the technique's utility. This quantitative approach provides clinicians with precise data to evaluate treatment effectiveness and plan subsequent clinical actions. However, the presented technique has certain limitations. The accuracy of spatial registration may be influenced by patient positioning during CBCT acquisition and minor anatomical changes unrelated to the lesion, potentially affecting the precise alignment of images (Alahmari et al. 2025). In particular, CBCT artifacts related to metallic restorations, dental implants, or prosthetic crowns may locally affect voxel intensity distributions, thereby influencing similarity metrics used for registration and potentially reducing the accuracy of image superimposition between scans (Schulze et al. 2011). Additionally, despite reducing observer variability, semi‐automatic segmentation still requires manual inputs, potentially introducing some residual subjectivity (Tian et al. 2024). In conclusion, the CBCT superimposition technique significantly improves diagnostic precision, consistency in longitudinal assessments, and overall clinical utility for radiographic monitoring of jawbone lesions, despite the identified limitations. Future developments could further enhance this method by integrating fully automated image analysis algorithms and machine learning techniques, potentially overcoming current limitations and optimizing clinical outcomes.

## 5 Conclusions

CBCT superimposition with semi‐automatic segmentation provides a fast, accurate, and reproducible tool for objectively tracking mandibular cyst regression, directly supporting clinical decisions in conservative management. Its reliability, however, hinges on consistent scan acquisition and still requires manual seed placement, highlighting the need for future automation to eliminate remaining user‐dependent variability.

## Author Contributions

**Francesco Fanelli:** image processing, volumetric measurements, and CBCT superimposition. **Angela Tisci:** manuscript drafting and revision. **Khrystyna Zhurakivska:** data interpretation and critical revision of the manuscript. **Giuseppe Troiano:** study conception and design, manuscript drafting, and final approval of the manuscript. All authors have read and approved the final version of the manuscript.

## Funding

The authors received no specific funding for this work.

## Ethics Statement

This study was conducted in accordance with the Declaration of Helsinki and all relevant national and institutional guidelines. The use of retrospective CBCT scans was reviewed and approved by the Local Ethics Committee of the “Policlinico Riuniti” University Hospital of Foggia (Ethical Approval No. 33/2025, granted on May 29, 2025).

## Consent

The requirement for written informed consent was waived by the Ethics Committee due to the retrospective nature of the study.

## Conflicts of Interest

The authors declare no conflicts of interest.

## Supporting information

Figure S1.

Figure S2.

## Data Availability

The authors have nothing to report.
