# Supplementary figures and images for: CBCT Image Superimposition for Longitudinal Monitoring of Mandibular Cyst Healing: A Technical Note
Source: Clin Exp Dent Res. 2026 Feb 18;12(2):e70292. doi: 10.1002/cre2.70292 (PMC12914340; doi:10.1002/cre2.70292)

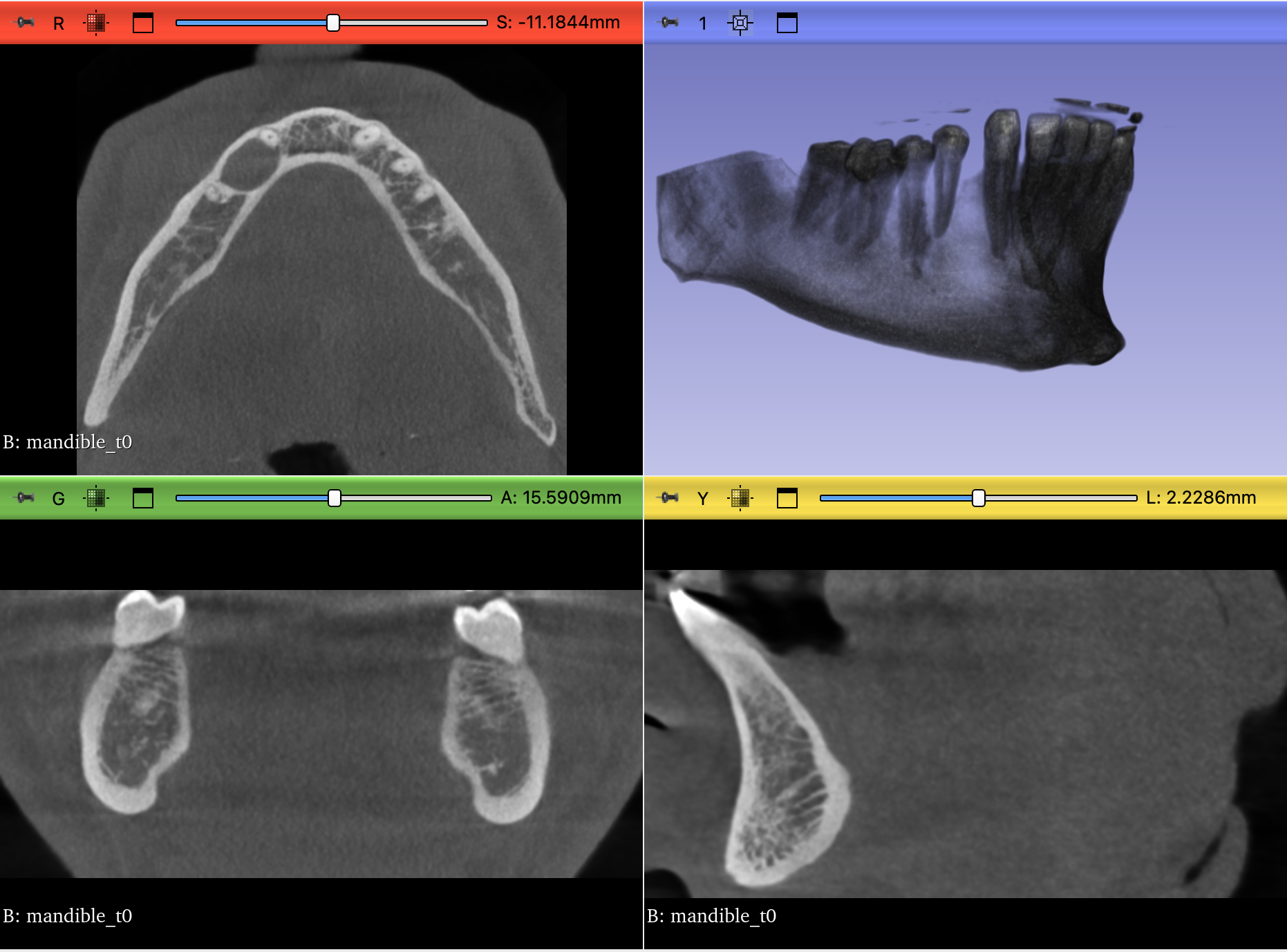

Supplement: Supplementary file 1 — Figure S1. [file CRE2-12-e70292-s002.jpeg]

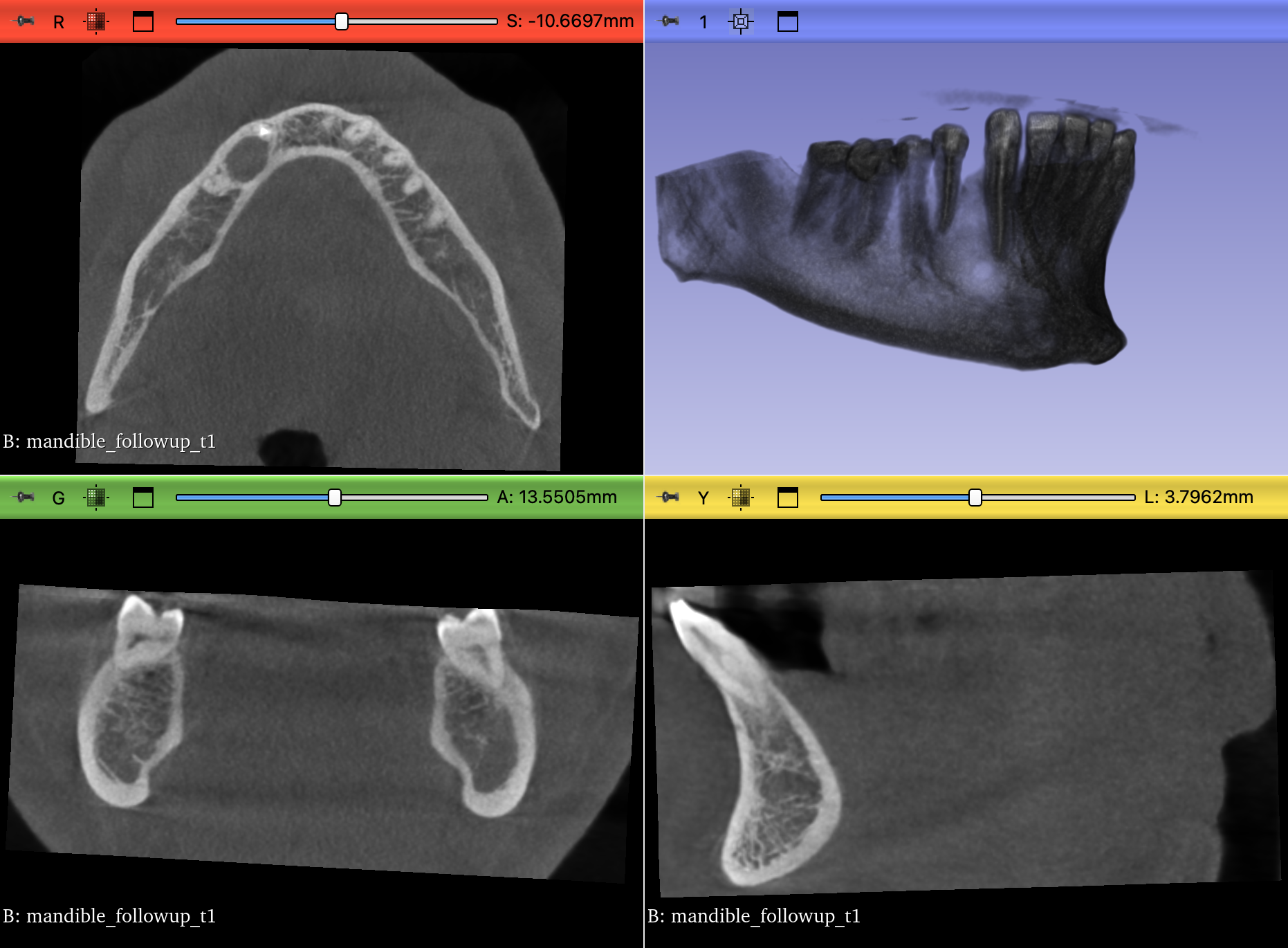

Supplement: Supplementary file 2 — Figure S2. [file CRE2-12-e70292-s001.jpeg]
